# Hemodynamic study: impact of anastomosis floor arterial stenosis on neointimal hyperplasia in hemodialysis arteriovenous fistulas

**DOI:** 10.3389/fbioe.2026.1706831

**Published:** 2026-07-10

**Authors:** Yanling Zhang, Ya Zhan, Yajun Xiao, Jiao Chen, Weihong Bi, Ting Wang, Jian Liu

**Affiliations:** 1 Department of Nephrology, The Affiliated Hospital, Southwest Medical University, Luzhou, Sichuan, China; 2 Department of Nephrology, The Third Hospital of Mianyang, Sichuan Mental Health Center, Mianyang, Sichuan, China; 3 Department of Geriatrics, The Third Hospital of Mianyang, Sichuan Mental Health Center, Mianyang, Sichuan, China; 4 Department of Endocrinology, The Third Hospital of Mianyang, Sichuan Mental Health Center, Mianyang, Sichuan, China; 5 The Affiliated Traditional Chinese Medicine Hospital, Southwest Medical University, Luzhou, Sichuan, China

**Keywords:** arteriovenous fistula, computational fluid dynamics, neointimal hyperplasia, oscillatory shear index, wall shear stress

## Abstract

**Objective:**

This study aimed to investigate the hemodynamic mechanisms associated with arterial stenosis at the anastomosis floor of arteriovenous fistula (AVF), with a particular focus on characterizing how varying degrees of stenosis affect hemodynamic parameters in downstream venous hyperplastic regions.

**Methods:**

Computational fluid dynamics (CFD) simulations were conducted to numerically analyze hemodynamic characteristics in AVF using a standardized idealized AVF model. Key hemodynamic parameters, such as wall shear stress (WSS) and oscillatory shear index (OSI), were quantitatively evaluated. Particular attention was given to the variations and spatial distribution of these parameters in the presence of stenotic lesions within the arterial wall at the anastomosis floor, as well as across varying severities of stenosis.

**Results:**

The regions of high wall shear stress in AVF are predominantly located at the anastomosis and along the outer wall of the juxta-anastomotic vein, whereas the low wall shear stress regions are primarily observed on the arterial wall at the floor of the anastomosis and along the inner wall of the swinging segment of the cephalic vein. These regions show partial consistency with the areas of high oscillatory shear index. In this idealized model, when the hyperplastic size of the artery at the anastomosis floor reaches approximately 1.0 mm, the wall shear stress level within the hyperplastic region returns to physiological levels, at which point neointimal hyperplasia ceases. At hyperplastic sizes of 0.5 mm and 1.0 mm in the artery at the anastomosis floor, the area of the high wall shear stress region on the outer wall of the juxta-anastomotic vein was reduced by 3.6% and 9.5%, respectively, compared to the non-hyperplastic state, with corresponding decreases in maximum wall shear stress of 7.0% and 10.2%. Similarly, under these hyperplastic conditions, the area of the low wall shear stress region on the inner wall of the swinging segment of the cephalic vein was reduced by 8.6% and 17.4%, respectively, relative to the non-hyperplastic state.

**Conclusion:**

In this idealized AVF model, when neointimal hyperplasia develops in the arterial wall at the anastomosis floor and reaches moderate severity, key hemodynamic parameters are restored toward physiological ranges, which is associated with attenuation of neointimal hyperplasia progression. This hyperplasia-induced moderate stenosis of the radial artery helps reduce the hyperplastic region in the cephalic vein near the anastomosis, thereby lowering the risk of stenosis and occlusion in the cephalic vein’s swing segment.

## Introduction

Hemodialysis represents the primary treatment modality for patients with end-stage renal disease (ESRD). Among available vascular access options, arteriovenous fistula (AVF) has emerged as the most commonly used access for maintenance hemodialysis, due to its distinct advantages, including a lower risk of infection, longer patency duration, and fewer complications (Gamé et al., 2025). However, studies indicate that the patency rate of AVF remains suboptimal, with an annual primary patency rate of approximately 70% (Liu et al., 2023; Yamazaki et al., 2020), thereby limiting its clinical effectiveness. AVF stenosis secondary to neointimal hyperplasia (NIH) has become one of the leading causes of AVF dysfunction, significantly compromising hemodialysis efficacy (Lok et al., 2020). As computational fluid dynamics (CFD) has advanced in medical applications, numerous researchers have utilized it for hemodynamic evaluation studies of AVF (Northrup et al., 2022; Wang et al., 2024; Poloni et al., 2025). It is well established that hemodynamic disturbances, which arise from significant flow alterations following AVF creation—such as disturbed flow, low wall shear stress (WSS), and oscillatory WSS—are key factors contributing to NIH and subsequent AVF failure (Hyde-Linaker et al., 2022; Colley et al., 2020; Shiu et al., 2019).

Hemodynamic studies on AVF have shown that regions prone to hyperplasia are primarily located at the arterial segment near the anastomosis floor, the inner wall of the cephalic vein’s swing segment (Ding et al., 2023; Soliveri et al., 2023; Ene-Iordache and Remuzzi, 2012; Yang et al., 2020). Ene-Iordache and Remuzzi (2012) were among the first researchers to identify two stenosis-prone regions in AVFs associated with low WSS and high oscillatory shear index (OSI): the artery floor and the inner wall of the juxta-anastomotic vein. Wang et al. (2024) also identified regions of low WSS at the floor of the anastomosis and the inner wall of the venous bending segment, in both modified and conventional AVF models using computational simulations. Soliveri et al. (2023) conducted a longitudinal study in which non-contrast magnetic resonance imaging and Doppler ultrasound (US) data were collected from a patient at multiple time points—3 days, 40 days, 6 months, 1 year, and 1.5 years—following AVF surgery. CFD simulations were performed, demonstrating that stenosis of the juxta-anastomotic cephalic vein developed at the 1.5-year follow-up, with the progression of stenosis accompanied by a corresponding decrease in near-wall disturbed flow indices. Chih-Yu Yang et al. conducted a CFD study involving 27 patients with angiographically confirmed stenosis. The results showed that stenotic lesions were predominantly located in the juxta-anastomotic cephalic vein, with the distance from the stenosis to the anastomosis measuring 21.9 ± 11.3 mm—consistent with the anatomical location of the cephalic vein’s swing segment (Yang et al., 2020). However, to date, no studies have investigated the progression patterns of arterial hyperplasia regions in AVF or their influence on the characteristics of downstream venous intimal hyperplasia. Given that arterial hyperplasia occurs upstream in the bloodstream, the vascular morphological changes induced by this hyperplasia are likely to affect the hemodynamic features and hyperplastic properties of the two downstream hyperplastic regions.

Using a standardized idealized AVF model, this study applies CFD simulation to systematically characterize hemodynamic alterations following AVF creation. Specifically, it aims to elucidate the hemodynamic mechanisms associated with arterial stenosis at the anastomosis floor of AVF and to investigate the effects of varying stenosis severities on hemodynamic parameters in downstream venous hyperplasia regions.

## Materials and methods

### Three-dimensional models of the AVF

**FIGURE 1:**
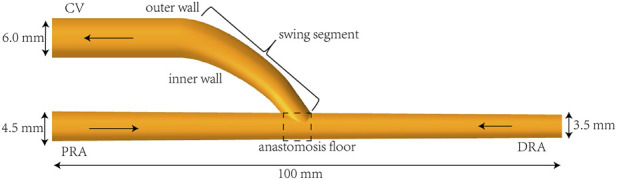
Idealized three-dimensional arteriovenous fistula model. PRA: proximal radial artery; DRA: distal radial artery; CV: cephalic vein.

The anatomical structure of the AVF has a significant impact on its hemodynamic behavior. To achieve a more accurate reconstruction of the three-dimensional geometry of the AVF, an idealized three-dimensional AVF model was developed based on ultrasound images (Supplementary Material 1) and data from a volunteer with a fully matured AVF. The resulting computational model is shown in Figure 1.

In the computational model, the artery is 100 mm in length, with a proximal diameter of 4.5 mm and a distal diameter of 3.5 mm; the arterial diameter tapers linearly from the proximal to the distal end. The anastomosis is located at the midpoint of the artery, forming a 45° angle and exhibiting an approximately elliptical shape (5.6 mm in length and 4 mm in width). The venous diameter increases rapidly from the anastomosis toward the downstream segment within the venous swing segment until it reaches 6 mm, after which it remains constant along the remaining length.

Computational models with varying severities of arterial stenosis at the anastomosis floor were developed, incorporating unilateral radial artery hyperplasia with thicknesses of 0.5 mm, 1.0 mm, and 1.5 mm. These models were utilized to analyze the effects of such stenosis on the NIH characteristics of AVF.

### Numerical methods

In the present study, the finite volume method was employed to simulate blood flow characteristics in AVF. For computational modeling, blood was approximated as a viscous, homogeneous, and incompressible Newtonian fluid, while gravitational effects were neglected. The governing equations used in the simulation included the continuity equation and the Navier-Stokes equations, which are presented as Equations 1, 2 below:

∇·u=0 (1)

$$\rho \left(\frac{\partial u}{\partial t}+u\cdot \nabla u\right)+\nabla p-\mu \nabla^{2}u=0$$ (2)

In the equations, *ρ* represents blood density with a value of 1,050 kg/m^3^; *t* denotes time; u is the velocity vector; *p* refers to pressure; and *μ* indicates the hydrodynamic viscosity coefficient of blood, which is assigned a value of 3.5  mPa s.

For the turbulence model, although the maximum Reynolds number at the proximal end reached 1,674, the *k*-*k*_*L*_-*ω* transition model was applied in this study. This selection was justified by the expectation that the elevated velocity at the anastomosis, combined with strong adverse pressure gradients and flow separation caused by vascular curvature, would induce flow transition and the formation of turbulent flow. The *k*-*k*_*L*_-*ω*model is particularly suitable for simulating transitional flows under conditions involving complex boundary layers and flow separation, and it provides a more accurate characterization of wall shear stress compared to laminar flow models.

### Computational grids and boundary conditions

A structured hexahedral grid was employed for the AVF model. Grid independence was verified using three grid densities: coarse (∼400,000 elements, first layer thickness = 0.006 mm), medium (∼640,000 elements, first layer thickness = 0.003 mm), and fine (∼920,000 elements, first layer thickness = 0.0015 mm). The area of low WSS regions was compared, and the maximum relative difference between medium and fine grids was less than 1.8%, confirming numerical convergence. The medium grid (640,000 elements) was selected for all subsequent simulations.

**FIGURE 2:**
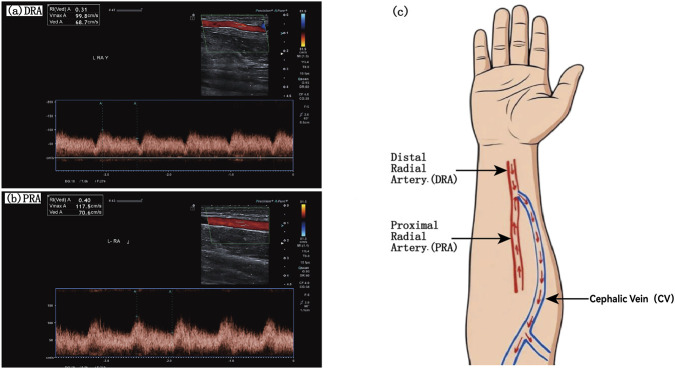
**(a)** Clinical measurements of blood flow velocities at the PRA and DRA of AVF using ultrasound. **(b)** Schematic of radial–cephalic AVF. **(c)** Schematic diagram of blood flow in arteriovenous fistula.

To enhance simulation accuracy, blood flow velocity data obtained from ultrasound imaging were assigned to the inlets of the proximal radial artery (PRA) and distal radial artery (DRA) of the arteriovenous fistula model, respectively. The peak systolic velocities were directly measured as 117.5 cm/s for PRA and 99.8 cm/s for DRA,as presented in Figure 2. The time-averaged velocity was calculated as 61.4% of the peak velocity according to the ratio of the clinically measured mean flow rate to the peak flow rate within one cardiac cycle. The inlet velocity used in simulations was set as a parabolic profile across the vessel cross-section. The inlet velocity waveform is presented in Supplementary Material 2.

A pressure outlet boundary condition was applied at the outlet of the AVF segment, with uniform pressure distribution across the entire cross-sectional area. A rigid no-slip wall boundary condition was was applied to the vascular walls.

During the computational process, a time step of 0.005 s was used. To ensure a stable periodic solution, the simulation results from the fourth cardiac cycle were used for data analysis.

The numerical method was verified by comparing the velocity magnitudes obtained from CFD simulations with those measured by clinical Doppler ultrasound at the proximal and distal radial artery of the AVF. The relative error between simulation and clinical measurement was less than 8%, confirming the reliability and accuracy of the present CFD model.

### Hemodynamic parameters

Time-averaged wall shear stress (TAWSS) and OSI were employed as hemodynamic parameters to assess the hyperplastic characteristics of AVF. TAWSS reflects the average wall shear stress over a single cardiac cycle. Its mathematical formulation is presented as follows:

$$\text{TAWSS}=\frac{1}{T}\int_{0}^{T}\vec{\left|WSS\right|}dt$$

Non-physiological WSS, which includes both low and high WSS, has been shown to disrupt endothelial function and induce NIH, thereby contributing to vascular stenosis (Chiu and Chien, 2011; Oka et al., 2020). In this study, consistent with the criteria widely adopted in relevant studies (Li et al., 2025; Malek et al., 1999), regions exhibiting shear stress values below 1 Pa were classified as low WSS zones, whereas areas with shear stress exceeding 10 Pa were categorized as high WSS zones.

OSI is a hemodynamic parameter that characterizes the temporal variation in the direction of WSS acting on the vascular intima. Its mathematical definition is expressed as:

$$\text{OSI}=0.5\left[1-\left(\frac{\left|\frac{1}{T}\int_{0}^{T}WSSdt\right|}{\frac{1}{T}\int_{0}^{T}\left|WSS\right|dt}\right)\right]$$

Clinical studies have consistently demonstrated that regions exposed to elevated OSI are particularly vulnerable to vascular endothelial injury, which can promote intimal hyperplasia through multiple pathways, including inflammatory responses, oxidative stress, and alterations in gene expression (Sorescu et al., 2004; Chappell et al., 1998; Meirson et al., 2015). Based on a comprehensive review of existing literature, the present study defines regions with OSI values exceeding 0.2 as high OSI zones (Lv et al., 2024; Tarrahi et al., 2020; Peiffer et al., 2013).

## Results

### Analysis of hemodynamic characteristics of AVF

**FIGURE 3:**
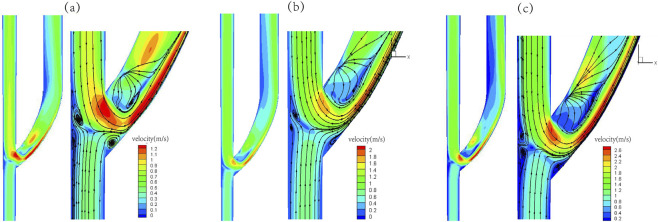
The blood flow velocity distributions in AVF at time points corresponding to the minimum, average, and maximum blood flow velocities. **(a)** Minimum blood flow velocities. **(b)** Average blood flow velocities. **(c)** Maximum blood flow velocities.

During the cardiac cycle, the blood flow velocity distributions in AVF are presented at time points corresponding to the maximum, average, and minimum blood flow velocities, respectively (as shown in Figure 3). Under each condition, the flow from the velocity inlet to the region near the anastomosis exhibits a characteristic laminar flow pattern. Near the anastomosis, the blood flows from both the proximal and distal ends of the artery deviate toward the anastomosis; upon convergence, the combined flow enters the vein. At the base of the anastomosis, within the radial artery, following the convergence of blood flows from the proximal and distal ends, a pair of separated vortices with opposite rotational directions are induced in their respective basal regions. The flow velocity within these vortices is significantly lower than that of the mainstream flow in the artery. In the anastomosis and its adjacent downstream region, an accelerated flow dominated by proximal blood flow is formed. Upon passing through the anastomosis, the high-velocity blood flow adheres closely to the outer wall of the cephalic vein, exhibiting a gradual deceleration trend; concurrently, an extensive low-velocity flow region develops along the inner wall of the cephalic vein. Two vortices are generated on both sides of the high-velocity flow, located at the inner edge of the anastomosis near the cephalic vein and within the region extending from the anastomotic opening to the distal end of the radial artery. These flow patterns are consistent with clinical observations and findings from previous studies (Colley et al., 2020; Li et al., 2025).

**FIGURE 4:**
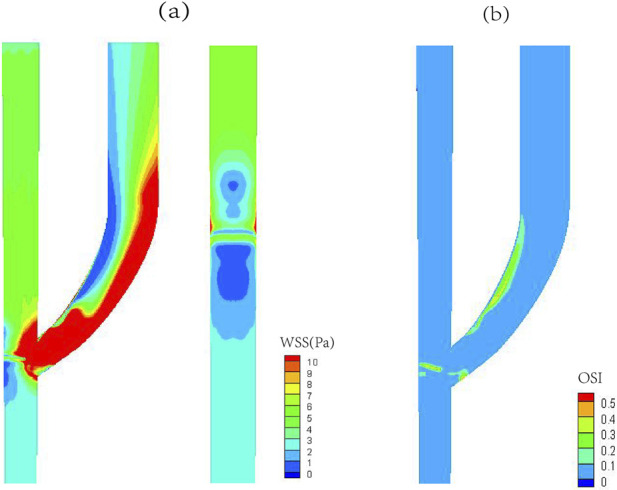
**(a)** Time-averaged wall shear stress distribution within the AVF. **(b)** Distribution of oscillatory shear index within the AVF.

Figure 4a illustrates the TAWSS distribution within the AVF. High wall shear stress regions (>10 Pa) are primarily observed at the anastomosis and along the outer wall of the juxta-anastomotic vein. Two distinct low wall shear stress regions (<1 Pa) are identified: one located at the radial artery near the anastomotic floor, and the other along the inner wall of the swing segment of the cephalic vein adjacent to the anastomosis. Notably, the low wall shear stress region on the inner wall of the cephalic vein swing segment is spatially consistent with the low-velocity flow region.

Figure 4b illustrates that within the AVF, regions exhibiting significant shear stress oscillations are located at the arterial wall of the anastomosis floor, the outer wall of the juxta-anastomotic vein, and the inner wall of the swing segment of the cephalic vein. Notably, the area of the high OSI region along the inner wall of the cephalic vein swing segment is considerably larger than those of the other two regions. This region is also recognized as a clinical hotspot for stenosis (Yang et al., 2020; Badero et al., 2008; Gunawardena and Ridgway, 2022).

### Analysis of arterial stenosis at the anastomosis floor on hemodynamic characteristics of AVF

**FIGURE 5:**
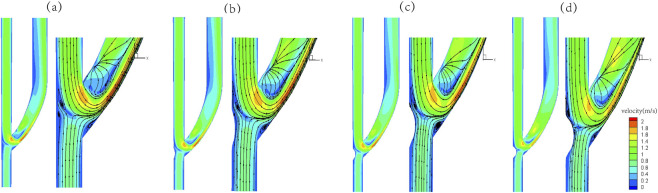
The velocity contour and streamline distribution at the time of average velocity under varying severities of stenosis at the anastomosis floor. **(a)** No stenosis. **(b)** 0.5 mm stenosis. **(c)** 1.0 mm stenosis. **(d)** 1.5 mm stenosis.

Figure 5 illustrates the velocity contour and streamline distribution on the symmetry plane at the average velocity instant under typical stenotic conditions. As the degree of arterial wall hyperplasia at the anastomosis floor increases, flow velocities at both the proximal and distal ends of the artery adjacent to the anastomosis also increase. When the hyperplasia thickness reaches 1.0 mm, the maximum flow velocity at the distal end increases from 0.7080 m/s to 0.7259 m/s. In contrast, the maximum velocity within the high-velocity flow region adjacent to the outer wall of the cephalic vein near the anastomosis decreases from 2.083 m/s to 2.016 m/s (refer to Table 1 for detailed data), accompanied by a slight reduction in the spatial extent of this high-velocity region. Simultaneously, the area occupied by the low-velocity flow region within the inner wall of the cephalic vein also decreases.

**TABLE 1:** Hemodynamic parameters of AVF under varying degrees of radial artery hyperplasia.

Hemodynamic parameters	0 mm	0.5 mm	1.0 mm	1.5 mm
Maximum velocity of anastomosis floor (m/s)	0.7080	0.7208	0.7259	0.7279
Maximum velocity of the outer wall of the juxta-anastomotic vein (m/s)	2.083	2.002	2.016	1.972
High WSS area in the outer wall of the juxta-anastomotic vein (mm^2^)	368.08	354.72	333.10	309.16
Low WSS area in the inner wall of the cephalic vein’s swing segment (mm^2^)	104.84	95.80	86.60	71.84
Maximum WSS value (Pa)	186	173	167	161

For the vortex located at the radial artery base adjacent to the anastomosis, neointimal hyperplasia on the arterial wall at the anastomosis floor leads to the hyperplastic surface being entirely covered by attached flow, remaining unaffected by separated vortices. The pair of separated vortices generated by the flow on the proximal and distal aspects of the artery are localized to the proximal root of the hyperplastic site. As the size of hyperplasia on the arterial wall at the anastomosis floor increases, the proximal separated vortex remains anchored at the root of the hyperplastic region, with neither its position nor its size being affected by the increase in hyperplasia size. In contrast, the distal vortex diminishes considerably with increasing hyperplasia size.

**FIGURE 6:**
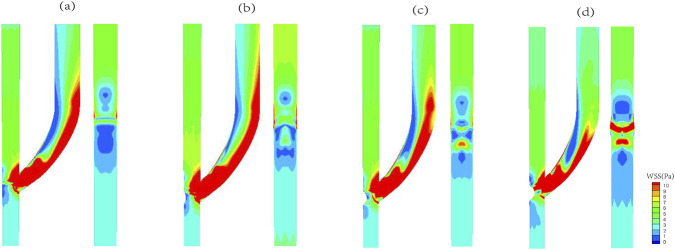
Time-averaged wall shear stress distribution in the AVF under varying severities of stenosis. **(a)** No stenosis. **(b)** 0.5 mm stenosis. **(c)** 1.0 mm stenosis. **(d)** 1.5 mm stenosis.

Figure 6 presents the TAWSS distribution of the AVF under typical stenotic conditions. As illustrated in the figure, with the development of hyperplasia at the anastomosis floor, Low wall shear stress regions are present at both the proximal and distal ends adjacent to the hyperplastic site, excluding the hyperplastic site itself. Among these, the low shear stress region at the distal end is notably smaller in area compared to that in the non-hyperplastic condition. The presence of hyperplasia alters the flow pattern in this region from separated flow (observed in the non-hyperplastic state) to attached flow, thereby significantly reducing the size of the low wall shear stress region. When hyperplasia occurs on the arterial wall at the anastomosis floor, only a few low wall shear stress regions are observed near the root area of the proximal end of the hyperplasia, and their locations are in close proximity to the separated vortices in this region.

The wall shear stress within the hyperplastic site itself increases with the growth of hyperplasia size. When the hyperplasia size is 0.5 mm or 1.0 mm, the proximal segment of the hyperplastic site corresponds to a normal wall shear stress region. At a size of 0.5 mm, the site’s own distal segment is dominated by low shear stress. As the hyperplasia size increases to 1.0 mm, its distal segment shifts to a normal shear stress dominance, with only an extremely small localized area of high shear stress. When the hyperplasia size reaches 1.5 mm, a large proportion of the hyperplastic site exhibits high wall shear stress, which will further accelerate hyperplastic progression.

According to the aforementioned developmental pattern, when the hyperplasia size approaches 1.0 mm, the entire hyperplastic region will maintain a normal wall shear stress level, leading to the cessation of neointimal hyperplasia. Consequently, within the geometric constraints of this model, arterial neointimal hyperplasia at the anastomosis floor is unlikely to progress beyond 1.0 mm.

When hyperplasia develops in the arterial wall at the anastomotic floor, extensive regions of high shear stress persist near the anastomosis and along the outer wall of the downstream cephalic vein. However, the spatial extent of the high shear stress region along the outer wall of the cephalic vein decreases in the direction of blood flow. At hyperplasia sizes of 0.5 mm and 1.0 mm, the area of this high shear stress region is reduced by 3.6% and 9.5%, respectively, compared to the non-hyperplastic condition, with corresponding reductions in maximum wall shear stress of 7.0% and 10.2% (refer to Table 1 for detailed data). Stenosis of the radial artery at the anastomotic floor effectively diminishes the extent of the high shear stress region along the outer wall of the cephalic vein near the anastomosis.

When hyperplasia develops in the arterial wall at the anastomosis floor, extensive regions of low shear stress persist along the inner wall of the cephalic vein swing segment. However, as the hyperplasia size increases, the spatial extent of these low wall shear stress regions decreases significantly in the direction of blood flow. At hyperplasia sizes of 0.5 mm and 1.0 mm, the area of low wall shear stress regions is reduced by 8.6% and 17.4%, respectively, both exceeding numerical uncertainty (<1.8%) and thus representing physically meaningful changes, compared to the non-hyperplastic condition (refer to Table 1 for detailed data). Stenosis of the radial artery at the anastomosis floor effectively reduces the extent of low wall shear stress regions on the inner wall of the cephalic vein swing segment.

**FIGURE 7:**
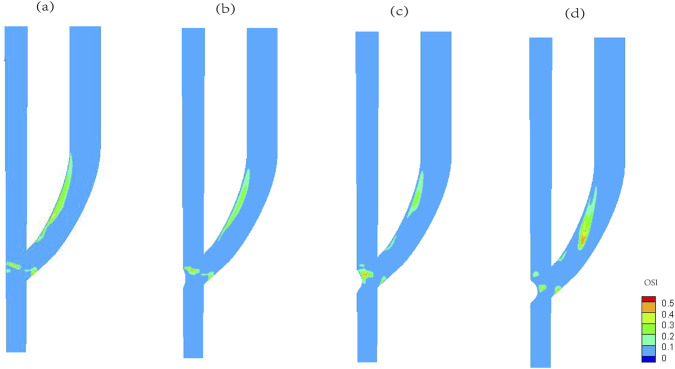
Oscillatory shear index distribution within the AVF under varying severities of stenosis. **(a)** No stenosis. **(b)** 0.5 mm stenosis. **(c)** 1.0 mm stenosis. **(d)** 1.5 mm stenosis.

Figure 7 presents the OSI distribution within the AVF under a representative stenotic condition. It can be observed that when intimal hyperplasia develops in the arterial segment at the anastomosis floor, neither the spatial distribution nor the magnitude of OSI exhibits notable alterations.

## Discussion

Currently, numerous hemodynamic studies on AVF have demonstrated that low WSS and elevated OSI are key hemodynamic factors associated with the induction of NIH (Wang et al., 2024; Hyde-Linaker et al., 2022; Ding et al., 2023; Malek et al., 1999; Williams et al., 2021; Cunnane et al., 2017). Building upon these findings, the present study investigates the characteristics of WSS and OSI in the vascular wall of AVF.

Analysis of WSS in AVF revealed that regions of high wall shear stress (>10 Pa) are predominantly localized at the anastomosis and along the outer wall of the swinging segment of the cephalic vein. These high-shear-stress regions exhibit a high degree of spatial correlation with high-velocity flow areas at and downstream of the anastomosis, and are closely related to the flow characteristics of high-velocity streams adhering to the vascular wall. This observation is largely consistent with the findings reported by Wang et al. (2024). The present study identified two distinct low-shear-stress regions (<1 Pa), among which the region located on the inner wall of the swinging segment of the cephalic vein demonstrates a significantly greater longitudinal extent and larger surface area compared to the low-shear-stress region in the radial artery at the anastomosis floor. This makes it theoretically the most susceptible region to NIH, which aligns with the widely accepted conclusion in current AVF intimal hyperplasia research that the inner wall of the swinging segment is a common site of stenosis (Gunawardena and Ridgway, 2022; Beathard et al., 2003).

Analysis of the OSI in AVF reveals that the shear oscillation region in the radial artery at the floor of the anastomosis coincides with the area characterized by varying reattachment lines between the two vortices. This region is formed due to the recurrent movement of the pair of vortices. The shear oscillation region on the outer wall of the juxta-anastomotic vein corresponds to the area influenced by the separated vortex at this location, which results from the vortex movement in the direction of blood flow. The shear oscillation region on the inner wall of the swinging segment of the cephalic vein exhibits the largest spatial extent among all identified regions. This region is marked by the cyclic formation and dissipation of local separated vortices and is situated within the core of the low-shear-stress zone, indicating that a substantial portion of this site demonstrates concurrent low WSS and high OSI characteristics. These hemodynamic features provide a mechanistic basis for the observation that the inner wall of the cephalic vein’s swinging segment is the most prevalent site of NIH.

**FIGURE 8:**
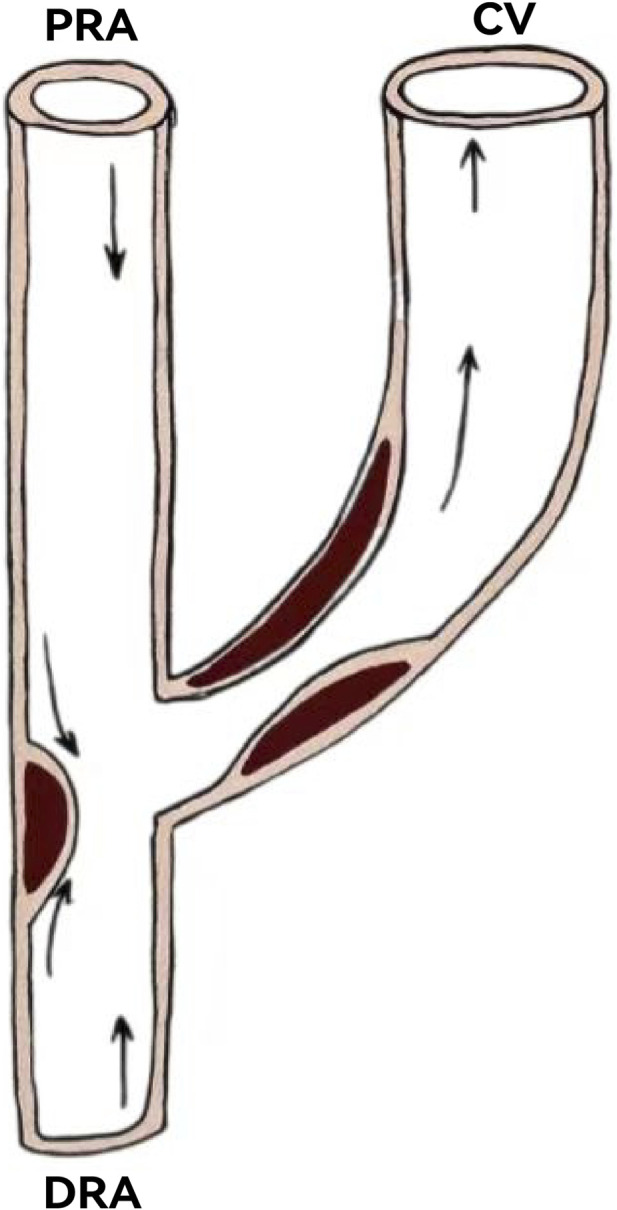
Schematic diagram illustrating the predilection sites for stenosis in AVF.

In summary, the artery at the anastomosis floor, the outer wall of the juxta-anastomotic cephalic vein, and the inner wall of the swinging segment of the cephalic vein represent high-incidence regions for NIH, with the inner wall of the swinging segment exhibiting the most pronounced manifestation of this pathology. This observation aligns with clinical findings reported in the literature (Yang et al., 2020). NIH in these regions is prone to progress to fistula stenosis. Based on the findings of this study, a schematic diagram illustrating the predilection sites for stenosis in AVF has been developed (as shown in Figure 8). This diagram shows some divergence from several previous studies (Ding et al., 2023; Cunnane et al., 2017; Sivanesan et al., 1999), which have identified the anastomotic base, the inner wall of the swinging segment, and the proximal vein at the straightening segment as the primary sites of stenosis. The key distinction in the present study is the identification of a more extensive, longitudinally distributed stenotic region along the inner wall of the swinging segment, extending into the straightening part of the proximal cephalic vein. This pattern is attributed to the widespread coexistence of low wall shear stress and high oscillatory shear index in this area. Furthermore, a limited region of elevated OSI exists on the outer wall of the juxta-anastomotic cephalic vein, where the WSS is significantly greater than 10 Pa, with localized values exceeding 150 Pa. Elevated WSS has been shown to contribute to vascular injury and subsequent stenosis through mechanisms such as mechanical endothelial damage, inflammatory responses, and endothelial dysfunction (Malik et al., 2022). Based on these findings, we propose that the outer wall of the juxta-anastomotic cephalic vein also represents a high-risk location for stenosis development.

Hemodynamic simulations based on an idealized AVF model demonstrate that the arterial segment at the anastomosis floor contains regions of low WSS and elevated OSI, which are conducive to the development of NIH and may ultimately contribute to stenosis at this anatomical site. As hyperplasia progresses within the anastomosis floor, a low WSS environment persists both proximally and distally adjacent to the hyperplastic region—suggesting the potential for lesion propagation along the axial direction and an increase in the longitudinal extent of radial artery involvement. However, the formation of stenosis leads to a reduction in local vascular cross-sectional area and an increase in blood flow velocity, which transforms separated flow into attached flow. This hemodynamic transition elevates local WSS to within the physiological range, thereby halting further hyperplastic progression. The dynamic interaction between vascular morphology and hemodynamic forces facilitates the establishment of a stable attached flow profile, enabling adaptive remodeling of vascular geometry in response to local flow conditions.

The present study demonstrates that, within the idealized model, when the proximal hyperplastic thickness of the lesion is within 1.0 mm and exhibits WSS within the physiological range, lesion progression is arrested. Similarly, when the distal hyperplastic thickness of the lesion is within the 0.5–1.0 mm range and a normal WSS distribution is established, further lesion growth is halted, ultimately resulting in a stable arterial stenosis at the anastomosis floor. Based on findings from the current idealized AVF model, the morphological characteristics of radial artery stenosis will eventually reach a steady state and will not culminate in vascular occlusion within this region. This 1.0 mm threshold is model-specific and may vary slightly with anatomical differences such as arterial diameter, anastomotic angle, and venous caliber across individual patients. Future patient-specific CFD and clinical imaging studies will be needed to validate the threshold in real-world anatomies.

In summary, the separated vortices generated by the convergence of blood flow from the distal and proximal segments of the radial artery within this region, together with their dynamic migration, represent a key mechanism driving the induction of NIH and subsequent stenosis. Conversely, stenosis within the arterial segment at the anastomosis floor improves hemodynamic properties by gradually transitioning separated flow to attached flow; concurrently, stenosis eliminates regions of low WSS through increased local blood flow velocity, ultimately effecting the cessation of hyperplasia.

It is widely acknowledged in existing literature that abnormal hemodynamic parameters in AVF can initiate NIH. In response to these disturbances, the hyperplastic site exhibits a compensatory proliferative tendency aimed at restoring disordered hemodynamic indices to physiological levels, which may ultimately result in luminal narrowing or occlusion (Soliveri et al., 2023; Remuzzi et al., 2017). However, clinical observations indicate that some AVFs remain functional for 5–10 years or longer without developing severe stenosis that compromises fistula performance. Based on this phenomenon, we propose the following hypothesis: Does a self-limiting equilibrium state exist that can halt the progression of intimal hyperplasia? Furthermore, could there be a form of NIH that serves a potentially adaptive or beneficial role?

The arterial stenosis zone at the anastomosis floor is situated upstream of the venous blood flow pathway; therefore, stenosis in this region directly influences the flow dynamics within the cephalic vein and alters its hemodynamic parameters. The present study demonstrates that arterial stenosis at the anastomosis floor exerts a notable modulatory effect on downstream flow characteristics. Specifically, such stenosis enhances the flow velocity in the distal segment, which subsequently reduces the velocity within the high-speed flow regions at and downstream of the anastomosis while increasing their distance from the vessel wall. This results in a reduction of WSS near these high-speed flow regions on the outer wall of the juxta-anastomotic cephalic vein and a decrease in the spatial extent of high WSS zones. Concurrently, the reduction in velocity within the high-speed flow regions leads to an increase in velocity within the low-speed flow regions on the inner wall of the swinging segment of the cephalic vein, thereby elevating local shear stress and reducing the area of low WSS regions.

From the perspective of the entire AVF system, although the change in OSI is not significant, small-scale local stenosis in the artery at the anastomosis floor can improve extensive regions of both high and low WSS within the cephalic vein. This improvement further suppresses NIH, reduces the total hyperplastic area across the AVF system, and thereby achieves overall optimization of the system. Meanwhile, clinical observations indicate that occlusion of the radial artery is relatively uncommon, whereas stenosis in the cephalic vein near the anastomosis is associated with a significantly higher risk of thrombosis, making it the primary contributor to fistula dysfunction (Hou et al., 2024; Marcinnò et al., 2024). Researchers suggest that with respect to the impact on AVF patency, more attention should be paid to the diameter of cephalic vein (Li et al., 2024). By enabling adaptive modifications in low-risk regions, the risk of occlusion in high-risk areas may be mitigated, leading to an overall reduction in system vulnerability and an improvement in functional performance.

Notably, the potentially beneficial effects observed herein were limited to moderate hyperplasia (0.5–1.0 mm). When hyperplasia reached 1.5 mm, pathological high WSS developed over the lesion surface, which would promote further vascular injury. Thus, only mild-to-moderate stenosis at the anastomotic floor serves a compensatory adaptive role, while severe stenosis becomes detrimental.

Clinically, these findings support a conservative surveillance strategy with monthly postoperative Doppler ultrasound follow-up to measure anastomotic floor artery hyperplasia. When hyperplasia is ≤1.0 mm (moderate adaptive stenosis), routine surveillance is sufficient. When hyperplasia exceeds 1.0–1.5 mm, it becomes pathological and may induce excessive wall shear stress injury; therefore, timely intervention (e.g., percutaneous angioplasty) is recommended to prevent detrimental progression.

In this model, arterial stenosis at the anastomosis floor is associated with reduced high and low WSS zones in the cephalic vein, which may mitigate NIH and lower occlusion risk. This phenomenon represents a form of NIH with potential adaptive benefits, reflecting an intrinsic self-optimization mechanism within the AVF system. Using CFD, this study elucidates the characteristics and beneficial effects of a type of potential NIH in clinical practice. Future research will focus on exploring strategies to translate this potentially beneficial physiological response into practical clinical applications.

## Conclusion

The arterial wall at the anastomosis floor, the outer wall of the juxta-anastomotic cephalic vein, and the inner wall of the swinging segment of the cephalic vein are high-incidence regions for NIH. In this idealized AVF model, when NIH in the arterial wall at the anastomosis floor reaches moderate severity, key hemodynamic parameters are restored toward physiological ranges, which is associated with attenuation of NIH progression. This hyperplasia-induced moderate radial artery stenosis is associated with improved venous hemodynamics that may lower the risk of stenosis and occlusion in the cephalic vein’s swing segment. These results reflect a potential adaptive mechanism rather than direct causal proof of clinical benefit.

## Data Availability

The original contributions presented in the study are included in the article/Supplementary Material, further inquiries can be directed to the corresponding authors.
