# Supplementary figures and images for: Hemodynamic study: impact of anastomosis floor arterial stenosis on neointimal hyperplasia in hemodialysis arteriovenous fistulas
Source: Front Bioeng Biotechnol. 2026 Jul 10;14:1706831. doi: 10.3389/fbioe.2026.1706831 (PMC13396247; doi:10.3389/fbioe.2026.1706831)

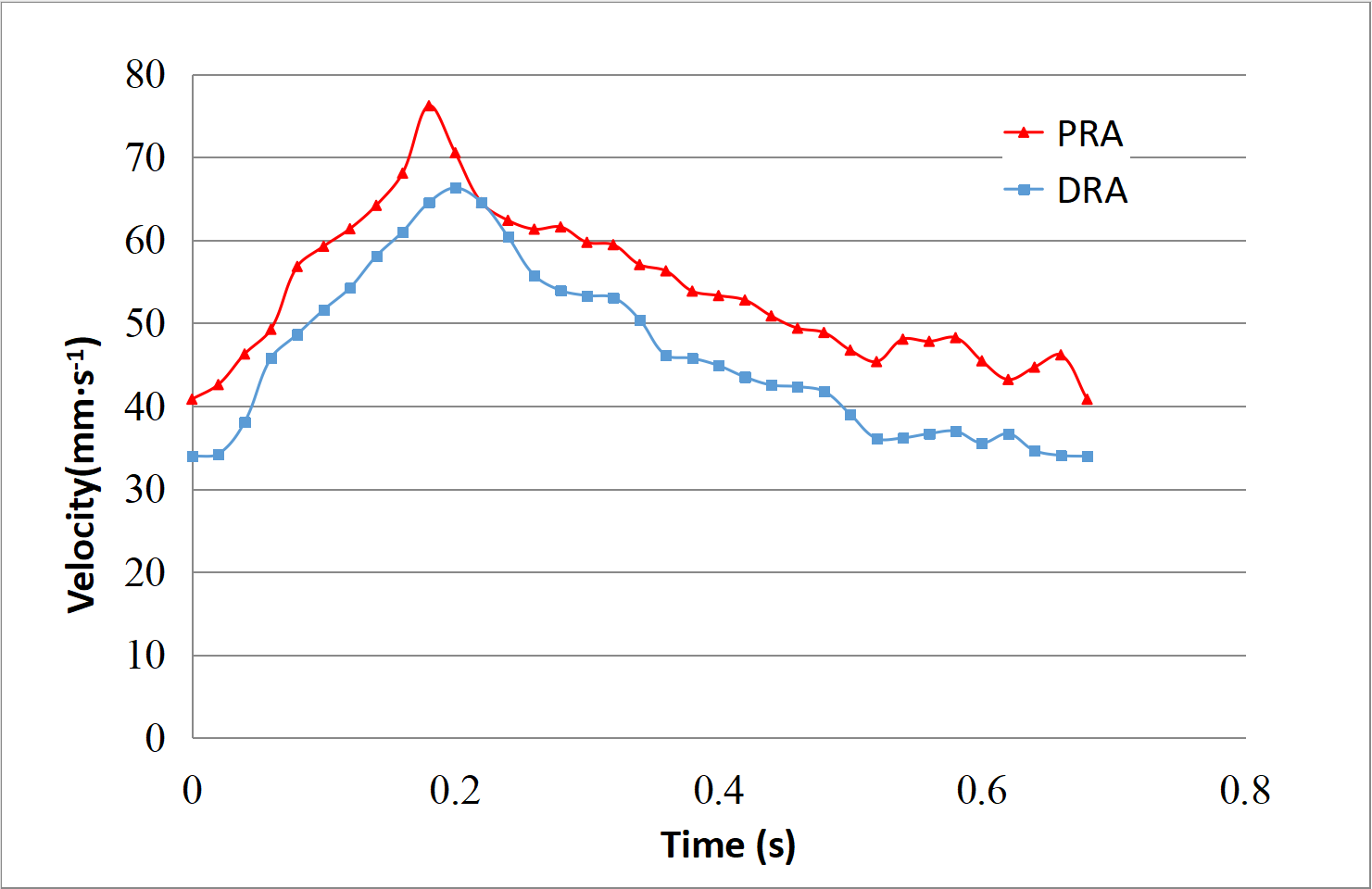

Supplement: Supplementary file 1 [file Image2.png]

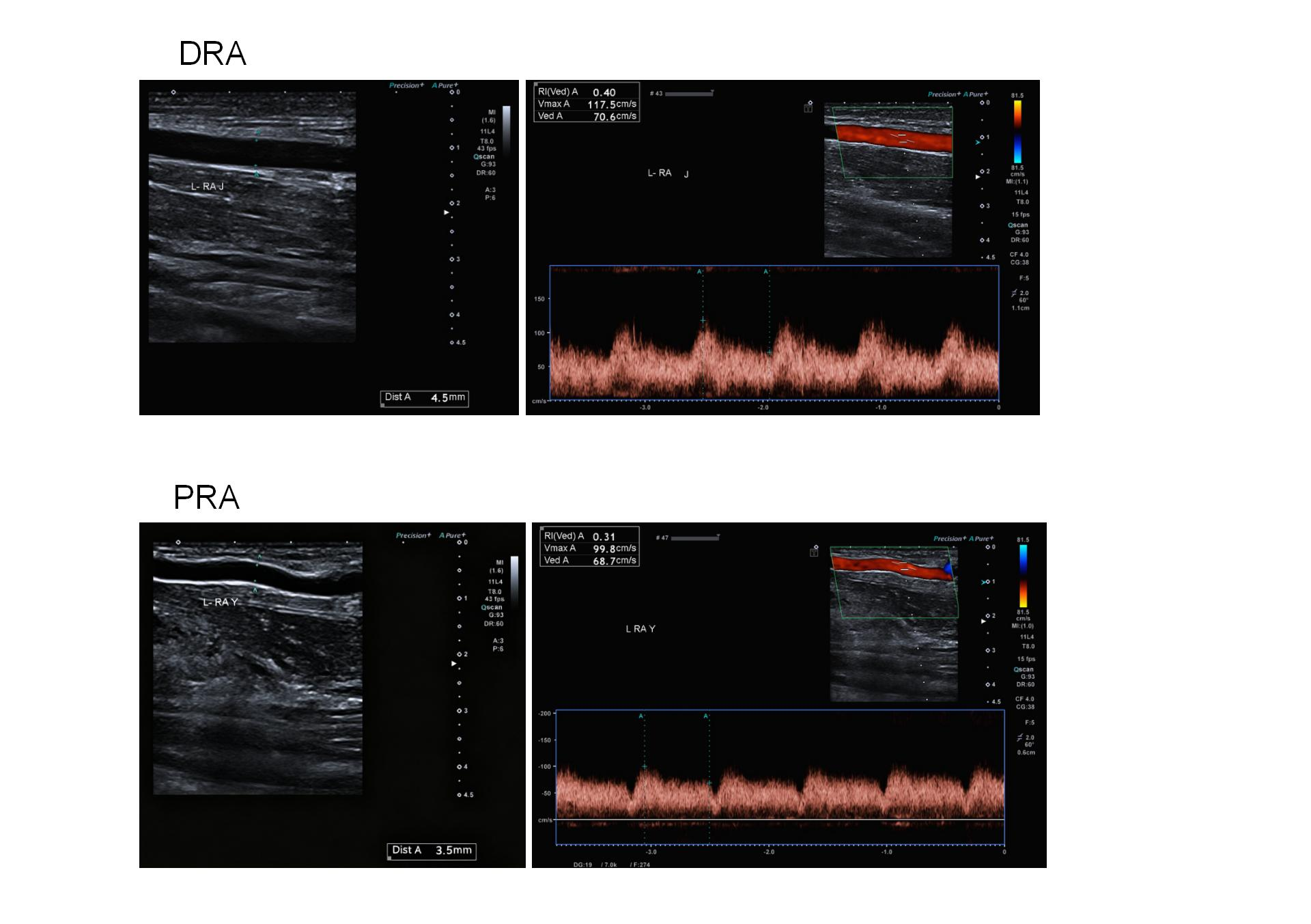

Supplement: Supplementary file 2 [file Image1.png]
